# The Association of Medication-Use and Frailty-Related Factors with Gait Performance in Older Patients

**DOI:** 10.1371/journal.pone.0149888

**Published:** 2016-02-22

**Authors:** Maartje H. de Groot, Jos P. C. M. van Campen, Nienke M. Kosse, Oscar J. de Vries, Jos H. Beijnen, Claudine J. C. Lamoth

**Affiliations:** 1 Department of Geriatric Medicine, MC Slotervaart, Amsterdam, The Netherlands; 2 Faculty of Health, Nutrition & Sport, The Hague University of Applied Sciences, The Hague, The Netherlands; 3 Center for Human Movement Sciences, University Medical Center Groningen, University of Groningen, Groningen, The Netherlands; 4 FRE 3405 AGIM Laboratory, CNRS-UJF-UPMF-EPHE, University Grenoble-Alpes, La Tronche, France; 5 Department of Internal Medicine, OLVG, Amsterdam, The Netherlands; 6 Department of Pharmacy & Pharmacology, MC Slotervaart, Amsterdam, The Netherlands; 7 Department of Pharmaceutical Sciences, Science Faculty, Utrecht University, Utrecht, The Netherlands; University of Tuebingen, GERMANY

## Abstract

The increased fall risk associated with the use of psychotropic drugs might be caused by underlying problems in postural control that are induced by sedative side-effects of these drugs. The current literature on the effects of psychotropics on postural control only examined acute single-drug effects, and included relatively healthy young elderly. Consequently, it is unclear what the impact of the long-term use of these drugs is on gait in frail older persons with polypharmacy. Therefore, it was aimed in the present study to explore the association between the use of psychotropics, multiple other medications, frailty-related parameters and gait performance in older patients. Eighty older persons (79±5.6 years) were recruited. Comorbid diseases, frailty-related parameters, and medication-use were registered. Trunk accelerations during a 3-minute-walking-task were recorded, whereof walking speed, mean stride times, coefficient of variation (CV) of stride times, and step consistency were determined. Multivariate Partial Least Squares (PLS) regression analysis was used to examine the association between population characteristics and medication-use, versus gait parameters. A PLS-model existing of four latent variables was built, explaining 45% of the variance in four gait parameters. Frailty-related factors, being female, and laxative-use were most strongly associated with lower walking speed, higher mean stride times, higher CV of stride times, and less consistent steps. In conclusion, frailty-related parameters were stronger associated with impaired gait performance than the use of psychotropic drugs. Possibly, at a certain frailty-level, the effect of the deterioration in physical functioning in frailty is so large, that the instability-provoking side-effects of psychotropic drugs have less impact on gait.

## Introduction

Older persons visiting a geriatric outpatient clinic are characterized by a combination of physical, psychological and social problems. They often have multiple chronic conditions, and use therefore multiple medications (polypharmacy). These frail old people have a high risk of adverse events, such as falling, hospital admissions, and ultimately death [1,2].

The past decades, many risk factors for falls in elderly people have been identified, which can be classified as either intrinsic (e.g., age-related changes in the sensor-motor system leading to gait and balance deficits), extrinsic (e.g., polypharmacy), or environmental factors [3]. In addition, meta-analyses showed that specific medication classes, including psychotropics and some cardiac drugs, have been associated with an increased risk of falling [4–7]. In a recent literature review [8], we suggested that the increased fall risk with which these psychotropic drug classes are associated, is caused by underlying problems in postural control these drugs can induce. That is because postural instability during daily activities, such as walking, is a primary cause of falling [9]. Walking can be characterized by a wide variety of gait parameters, each characterizing different aspects of the gait pattern. Walking speed is easily assessable and often used in clinical practice to reflect the functional status in older patients [10]. However, it lacks specificity (gait speed is slower in many different pathologies), and does not reveal information about the quality of the walking pattern, i.e., the ability of a person to adapt his/her gait smoothly, and the variability and regularity of the gait pattern. Stride-to-stride variability during walking provides additional information about gait performance, since high variability in stride times in frail elderly can be considered as a marker of abnormal regulation of gait [11,12]. Both walking speed and characteristics indicating the variability in the walking pattern are suggested to be associated with increased fall risk [13,14].

The current literature about the effects of psychotropic drugs on postural control assessed postural control during quiet standing, described only acute single-drug effects, and included relatively healthy young elderly [8]. Thus, the impact of long-term psychotropic drug-use on gait performance in frail older people with multiple comorbid diseases and polypharmacy remains still unclear.

In the present study, we therefore aimed to explore the association between the use of psychotropic drugs and gait performance in a cohort of older patients. Because in this frail older population many factors may be simultaneously present that could influence gait (e.g., the use of multiple medications, the presence of comorbid diseases, cognitive impairment, and/or frailty [11,12,15,16]), we included these factors in our analyses as well. The use of a cross-sectional study design, and a Partial Least Squares (PLS) regression analysis, enabled us to examine the association between the use of psychotropic drugs and gait parameters, in relation to other factors that might influence the walking pattern. We hypothesized that a deterioration in gait performance was not only explained by the use of psychotropics, but that in older patients frailty-related parameters, polypharmacy and comorbidity would also be associated.

## Methods

### Ethics Statement

The study was approved by the local Medical Ethics Committee of the MC Slotervaart (registration number: NL33825.048.10). All participants or their legal representatives (when participants had cognitive impairment) gave their written informed consent.

### Participants

Eighty patients were recruited consecutively among the elderly that visited the geriatric outpatient clinic of the MC Slotervaart in Amsterdam between October 2010 and April 2013. They were referred to the clinic by their general practitioner for various reasons, including memory complaints, mobility problems, or for evaluating polypharmacy. Patients were eligible to participate in the study if they were at least 65 years or older, and could walk safely for at least 3-minutes without any assistive device. They were excluded when they had any mobility problems due to neurological or orthopedic disorders, or were not able to understand the instructions of the researcher due to severe cognitive or hearing impairment.

During the study period, 619 older adults visited the geriatric outpatient clinic. Thereof, 392 patients (63%) did not meet the in- and exclusion criteria. In total, 227 (37%) patients were eligible, whereof 80 patients (13% of all visitors of the geriatric outpatient clinic) were willing to cooperate in the present study. The other 147 patients refused to cooperate because they felt too tired after the day in the hospital or did not want to cooperate (without giving reason).

### Measurements

#### Population characteristics

All participants underwent extensive physical and cognitive examination, as part of a Comprehensive Geriatric Assessment (CGA) [17]. General information was obtained from their patient file. All population characteristics were binary coded for statistical analyses (see S1 Dataset). Age was coded as being ≥80 or <80 years, because this was the mean age of the included participants). Cognitive functioning was assessed using the Mini Mental State Examination (MMSE) [18]. Participants scoring ≤23 points were categorized as being cognitive impaired [19]. Fall risk was examined using the LASA fall risk assessment [20]. When participants scored ≥8 points, they were identified as having an increased risk of recurrent falling. Four frailty criteria were registered [1], namely: unintentional weight loss, self-reported exhaustion, low physical activity, and low hand grip strength. Walking speed was a dependent variable in the statistical analyses. Comorbid diseases were registered using the Charlson’s Comorbidity Index (CCI) [21]. Table 1 lists the registered comorbid diseases.

**Table 1:** Prevalence of the independent variables (population characteristics, comorbid diseases, and medication-use per ATC-drug class) is presented in the left column as *n* (%) in the population (N = 80). In the right column, the captured variance (%) in every independent variable per latent variable (LV) of the PLS-model, and the total variance is presented. Values with high modeling power (>6.90%)^a^ are relevant to the LVs.

		Prevalence		Captured Variance (%)				
Independent variables		*n*	(%)	LV 1	LV 2	LV 3	LV 4	TOTAL
*Population characteristics*								
Female		50	(63%)	13.26	3.20	24.72	2.37	43.55
≥80 years		41	(51%)	9.65	0.75	1.94	3.41	15.76
≥2 comorbid diseases		29	(36%)	13.21	16.63	4.86	6.84	41.55
Cognitive impairment (MMSE ≤23 points)		35	(44%)	0.55	2.70	0.24	3.22	6.71
Increased fall risk (Pluijm score ≥8 points)		14	(18%)	15.90	1.09	21.45	0.82	39.26
Polypharmacy (≥4 medications)		52	(65%)	35.52	20.44	5.44	1.26	62.66
Frailty-criteria								
- Unintentional weight loss		13	(16%)	14.09	3.30	1.78	1.83	20.99
- Self-reported exhaustion		21	(26%)	24.77	0.00	0.44	1.67	26.87
- Low physical activity		12	(15%)	24.92	0.23	0.05	2.18	27.38
- Low hand grip strength		20	(25%)	10.85	1.33	24.00	5.71	41.89
*Comorbid diseases (CCI)*								
Myocardial infarct		21	(26%)	9.51	11.14	0.12	19.40	40.17
Peripheral vascular disease		8	(10%)	2.39	6.85	2.03	0.05	11.32
Cerebrovascular disease		5	(6%)	0.39	0.02	0.00	0.21	0.62
Dementia		22	(28%)	0.37	2.90	10.44	1.74	15.45
Chronic pulmonary disease		8	(10%)	7.45	0.45	19.96	2.32	30.17
Connective tissue disease		2	(3%)	0.00	0.65	1.28	0.06	1.99
Ulcer disease		4	(5%)	3.96	0.31	0.37	6.39	11.02
Diabetes		11	(14%)	2.92	4.67	0.69	0.36	8.65
Moderate or severe renal disease		6	(8%)	2.81	11.53	1.22	1.96	17.52
Diabetes with end organ damage		2	(3%)	3.35	0.48	0.50	3.51	7.84
Any tumor		10	(13%)	0.81	8.31	0.74	6.66	16.52
Leukemia		1	(1%)	0.49	0.22	1.11	0.01	1.83
*Medication-use (per ATC-drug class)*								
Agents for alimentary tract & metabolism (group A)								
A02A	Antacids	2	(3%)	2.01	0.55	4.25	0.13	6.93
A02B	Drugs for peptic ulcer and gastro-oesophageal reflux disease (GORD)	31	(39%)	39.46	7.92	0.21	0.66	48.24
A03	Drugs for functional gastrointestinal disorders	3	(4%)	4.76	0.00	3.24	2.15	10.15
A06	Drugs for constipation	12	(15%)	11.13	4.29	6.06	0.65	22.13
A07	Antidiarrheals, intestinal anti-inflammatory/anti-infective agents	2	(3%)	4.33	0.08	1.79	0.04	6.24
A09	Digestives, incl. enzymes	1	(1%)	2.25	0.17	3.91	0.38	6.71
A10	Drugs used in diabetes	11	(14%)	5.63	8.89	1.40	0.01	15.92
A11 & A12	Vitamins & mineral supplements	25	(31%)	9.07	0.63	18.18	2.43	30.30
Drugs acting on the cardiovascular system (group C)								
C01AA05^a^	Digoxin	4	(5%)	0.03	1.26	0.13	0.98	2.41
C01B	Antiarrhythmics (class I and III), excl. type IA	2	(3%)	0.61	0.37	3.46	0.76	5.20
C01BA	Type IA antiarrhythmics^b^	0	(0%)^b^					
C01D	Vasodilators used in cardiac diseases	10	(13%)	6.79	16.39	0.96	2.50	26.65
C01E	Other cardiac preparations	2	(3%)	1.03	3.91	0.84	1.59	7.37
C03	Diuretics	30	(38%)	2.01	27.48	6.21	1.61	37.31
C07	Beta blocking agents	25	(31%)	1.55	18.13	3.25	10.40	33.33
C08	Calcium channel blockers	13	(16%)	4.52	9.03	0.00	1.76	15.31
C09	Agents acting on the renin-angiotensin system	31	(39%)	0.56	35.73	4.91	13.54	54.74
C10	Lipid modifying agents	38	(48%)	3.98	20.24	7.59	4.82	36.64
Drugs acting on the nervous system (group N)								
N02	Analgesics (no paracetamol)	6	(8%)	12.15	0.26	7.85	2.06	22.33
N02BE	Paracetamol/acetaminophen	5	(6%)	9.95	0.10	15.57	2.74	28.37
N03A	Antiepileptics	6	(8%)	1.12	1.81	7.29	4.23	14.46
N05A	Antipsychotics	2	(3%)	1.61	0.68	0.01	0.21	2.52
N05BA	Anxiolytics (benzodiazepine-derivatives)	8	(10%)	0.47	0.11	0.00	0.83	1.41
N05C	Hypnotics, excl. benzodiazepine-derivatives	7	(9%)	15.58	1.61	5.70	2.91	25.80
N05CD	Hypnotics & sedatives (benzodiazepine-derivatives)	5	(6%)	0.37	0.44	4.20	0.00	5.02
N06A	Antidepressants	12	(15%)	7.93	7.15	0.01	2.07	17.15
N07	Other nervous system drugs	2	(3%)	0.24	0.47	6.05	0.03	6.80
Other ATC-drug classes								
B01	Antithrombotic agents	34	(43%)	12.48	22.64	7.16	2.30	44.59
B03	Anti-anemic preparations	4	(5%)	3.67	1.56	0.41	6.58	12.22
D	Antifungals for dermatological use	1	(1%)	2.76	2.46	2.35	0.04	7.61
G	Genito-urinary system and sex hormones	11	(14%)	12.63	1.39	0.73	2.04	16.78
H	Systemic hormonal preparations, excl. sex hormones and insulins	5	(6%)	7.85	0.61	1.03	1.51	10.99
J	Anti-infectives for systemic use	4	(5%)	5.15	0.38	0.07	2.77	8.37
L	Antineoplastic and immunomodulating agents	1	(1%)	0.01	2.78	0.83	6.49	10.11
M	Musculo-skeletal system	10	(13%)	5.88	1.59	0.61	6.38	14.46
R	Respiratory system	13	(16%)	7.71	0.02	5.77	0.82	14.32
S	Sensory organs	8	(10%)	3.41	17.45	5.68	5.63	32.18

^a^ Variables with low modeling power, i.e., around A/K, are of little relevance (A = number of LVs = 4; and K = number of independent variables = 58). Thus variables with less variance captured than 4/58 = 6.90% are not important to the LV [22].

^b^ This item was excluded from further PLS-analyses, because n = 0.

#### Medication-use

Medication-use was systematically reviewed by the participant’s physician as part of the CGA. Prior to their visit to the Geriatric Outpatient Clinic, patients were requested to bring the packaging of their medications or a list of their pharmacist with the drugs they use. The physician went through the list with the patients and/or their caregivers to check if the patients used all medications appropriately and/or used other non-prescribed drugs or supplements. The actual medication-use was then registered in the patients’ file, which was used in the present study.

All generic names of the drugs (trade names) were manually looked up and subsequently linked with the corresponding code of Anatomical Therapeutic Chemical (ATC) classification system [23] (see S1 Table for an overview of the prevalence of all drugs in the study population). The medications were clustered into ATC-drug classes (see Table 1 for an overview of the 37 included ATC-drug classes in the present study). For each participant it was binary scored whether the participant used a drug in a specific ATC-drug class or not (see S1 Dataset). Furthermore, the total number of drugs used per participant was registered. Polypharmacy was present when the participant used ≥4 drugs.

#### Gait parameters

Participants walked 3-minutes in a 80-meter long hallway at a self-selected speed while trunk accelerations were recorded using an accelerometer (DynaPort Minimod Hybrid; McRoberts BV; sample frequency 100 Hz), which was attached with a band at the level of the lumbar spine. Walking distance was recorded to determine gait speed. Trunk acceleration signals were analyzed using custom-made software in MATLAB (version 2011b; The MathWorks Inc.). Foot contact moments were determined from the peaks of the anterior-posterior-acceleration time-series. From the foot contact moments, stride times were calculated defined as the time interval between two ipsilateral foot contacts. For each participant, mean and CV of stride times were computed. The variability between left and right steps within the strides was quantified by the standard deviation (SD) of the relative timing between ipsilateral foot contact moments, with larger values indicating less consistent steps [24,25]. See De Groot et al. [16] for a detailed description of the calculated gait parameters.

### Statistical Analyses

To test the association between the various drug classes, the population characteristics, and the comorbid diseases in relation to stride variability, a multivariate PLS regression analysis was computed using the PLS_toolbox for Matlab (version 3.7.1; Eigenvector Research Inc.). PLS-regression is a technique that combines features from principal component analysis and multiple regression, and is not impeded by collinearity among variables [26–28]. PLS has particularly been applied in studies in which a set of dependent variables is predicted from a relatively large set of independent variables with relatively few observations [29–31]. In these studies, similar to our study, continuous, ordinal and binary data were included. It is well known that their exists an interdependency among different gait variables, such as walking speed, mean stride times, and variability in stride times [32–34]. When using PLS, a model can be constructed wherein the dependency among these gait variables is taken into account. That makes the PLS-regression analysis more favorable over the more conventional multiple regression analysis.

In the PLS-regression analysis, 58 independent variables (the population characteristics, comorbid diseases, and medication-use) and four dependent gait parameters were included. In order to give the variables the same scale, i.e., the same importance in the analysis, all independent (binary coded) variables were mean-centered (i.e., means of each column were set to zero), and the dependent gait parameters were scaled to unit variance by dividing each variable by their SD’s and centering them by subtracting their averages [22,35].

PLS-regression analysis searches for a set of latent variables (LVs) explaining as much as possible of the covariance between the independent (the population characteristics, comorbid diseases, and medication-use per ATC-class), and dependent variables (the four gait parameters). With numerous and correlated variables, there is a substantial risk for “over-fitting”, i.e., getting a well-fitting model with little or no predictive power. Cross-validation is a practical and reliable way to test the predictive significance of the model [35]. Cross-validation was performed by dividing the data into eight groups (method: Venetian Blinds), and then developing eight parallel models from reduced data with one of the groups deleted. Then, differences between the actual and the predicted values for the gait parameters were calculated for the deleted data. The sums of squares of these differences were computed and collected from all the parallel models to form the predictive residual sum of squares (PRESS), which estimates the predictive ability of the model. The optimal number of LVs was determined by stop adding LVs as soon as the Predicted REsidual Sum of Squares (PRESS) decreased [36]. From the PRESS-values of the final model, the predictive ability of the model was indicted by the parameter Q^2^, the predicted variation, or: *the goodness of prediction*. This value can be compared to R^2^, the explained variation, which is the capacity of the population characteristics, comorbid diseases and medication-use to explain the variance among the gait variables, or: *the goodness of fit*.

From the PLS model, a set of model parameters was computed (see Table 2). A downside of using PLS is that it is relatively difficult to interpret model parameters, since these are biased and *p*-values and confidence intervals are lacking. By interpreting the model parameters simultaneously, the associations between the independent variables (the population characteristics, comorbid diseases, and medication-use) were examined in relation to the gait parameters. The relevance of each independent variable to the LVs was indicated by the percentage variance that was captured by the LV [22]. Variables with low modeling power, i.e., around A/K (in our study, A = number of LVs = 4; and K = number of independent variables = 58), were of little relevance. Thus variables with variance below 4/58 = 6.90% are not important to the LV [22,29]. Therefore, variables with high captured variance for the same LV were clustered in the same LV.

**Table 2:** List of abbreviations and explanations of outcome variables of the PLS-analysis.

Abbreviation	Outcome variable	Indicator of	Interpretation
	Independent variables		Population characteristics, comorbid diseases, medication-use in various ATC-drug classes
	Dependent variables		Gait parameters: walking speed, mean stride times, CV of stride times, and step consistency
Q^2^	Predicted variance	Goodness of prediction	Higher Q^2^ means better predictive ability of the model. Q^2^ >50% is regarded good [22].
R^2^	Explained variance	Goodness of fit	Higher R^2^ means better capacity of the independent variables to explain the variance among the gait parameters
RC	Regression Coefficient	Association between independent and dependent variable in the model	Higher positive or negative RC means that the independent variable is stronger related to gait parameter
	Variance Captured	Relevance of the independent variable to the LV	Variance>6.9% means that independent variable is relevant to the LV. Higher captured variance means more relevant
VIP	Variable Importance on Projection	Importance of each independent variable for the gait parameters	VIP>1.0 means that independent variable is influential to the gait parameters. Higher VIP means more influence

The association between each independent variable and the gait parameters was indicated by regression coefficients. A higher positive or negative regression coefficient means that the independent variable is stronger related to the gait parameter. The importance of each variable in explaining the variation among gait parameters was given by the variable importance in projection (VIP) value. A VIP-value higher than 1 means that the independent variable is influential to the gait parameters. At last, score and weight plots illustrate and summarize the relationship between observations (the participants) and independent variables (population characteristics, comorbid diseases and medication-use), respectively, with respect to the latent variables.

Since the model parameters are biased, the important variables in relation to the gait parameters have overall higher modeling power (high captured variance), have higher positive or lower negative regression coefficients, and have a VIP-value higher than one. Moreover, these important variables have higher positive or lower negative weight-values, and are therefore clustered in the upper right or lower left quadrant of the weight plot. Based on the scores and weights of the model, predicted values for the gait parameters were calculated to illustrate the fit of the model [22,28,36]. See for a more detailed mathematical description of the PLS regression analysis the Supporting Information (S1 Appendix).

## Results

### Descriptive Characteristics of the Study Population

Eighty older persons were included in the present study (aged 79±5.6 years). Cognitive impairment was present in 35 participants (44%); the median MMSE score was 24 points (range: 11–30). Fourteen participants (18%) scored ≥8 points on the LASA fall risk assessment, indicating an increased risk for recurrent falls. Considering the frailty-related indicators, 13 participants (16%) reported ≥5kg unintentional weight loss, 21 participants (26%) had self-reported exhaustion, 12 participants (15%) had low physical activity, and 20 participants (25%) had low grip strength. Twenty-nine participants (36%) had ≥2 comorbid diseases on the CCI. Dementia, myocardial infarct, and diabetes were most present in the included population (respectively 27%, 26%, and 14%). See Table 1 for the prevalence of other comorbid diseases.

The participants used 5.5±3.9 drugs; 52 participants (65%) were categorized as having polypharmacy, because they used ≥4 drugs. Twenty-nine participants (36%) used one or more psychotropic medications that are known to be associated with increased fall risk, e.g. antiepileptics (N03A), antipsychotics (N05A), anxiolytics (N05BA), hypnotics (N05C), and antidepressants (N06A). Medication-use per ATC-class is presented in Table 1.

Mean walking speed was 0.90±0.24 m/s, mean stride time was 1.16±0.13 seconds, the variation (CV) of stride times was 3.81±1.61%, and step consistency was 4.67±1.68°.

### Association Between Population Characteristics, Comorbid Diseases, and Medication-Use in Relation to Gait Parameters

The PLS-regression testing the association between population characteristics, comorbid diseases and medication-use in relation to gait parameters yielded a model of four LVs (see Table 3), explaining 27.6% of the variance between the population characteristics, comorbid diseases, and medication-use, and 45.4% of the variance in gait parameters (R^2^). Adding more LVs to the model did not improve R^2^, as indicated by the PRESS.

**Table 3:** Explained variance (%) in the population characteristics, comorbid diseases and medication-use (independent variables) and gait parameters (dependent variables) by the PLS-model existing of four latent variables (LVs).

	LV 1	LV 2	LV 3	LV 4	TOTAL
Explained variance in the independent variables (%)	9.62	8.57	5.76	3.67	27.61
Explained variance in the dependent variables (%)	20.71	9.33	5.70	9.69	45.43

The variable most relevant to the model, i.e., with the most variance captured by the model, was the presence of polypharmacy (63%). The first LV captured most variance of the following variables: using drugs for ulcer and reflux disease (A02B; 39%), polypharmacy (36%), and two frailty criteria: low physical activity (25%), and self-reported exhaustion (25%). The second LV captured most variance in polypharmacy (20%), and in using drugs acting on the cardiovascular system (ATC-drug classes C09, C10, C03, C07, and C01D). Most variance by the third LV was captured in being female (25%), having low hand grip strength (24%), and increased fall risk (21%). By the fourth LV, most variance was captured in having myocardial infarction in medical history (19%), and using agents acting on the renin-angiotensin system (C09; 14%), and beta-blocking agents (C07; 10%). The variance captured by the model for all variables per LV are presented in Table 1.

**Fig 1:**
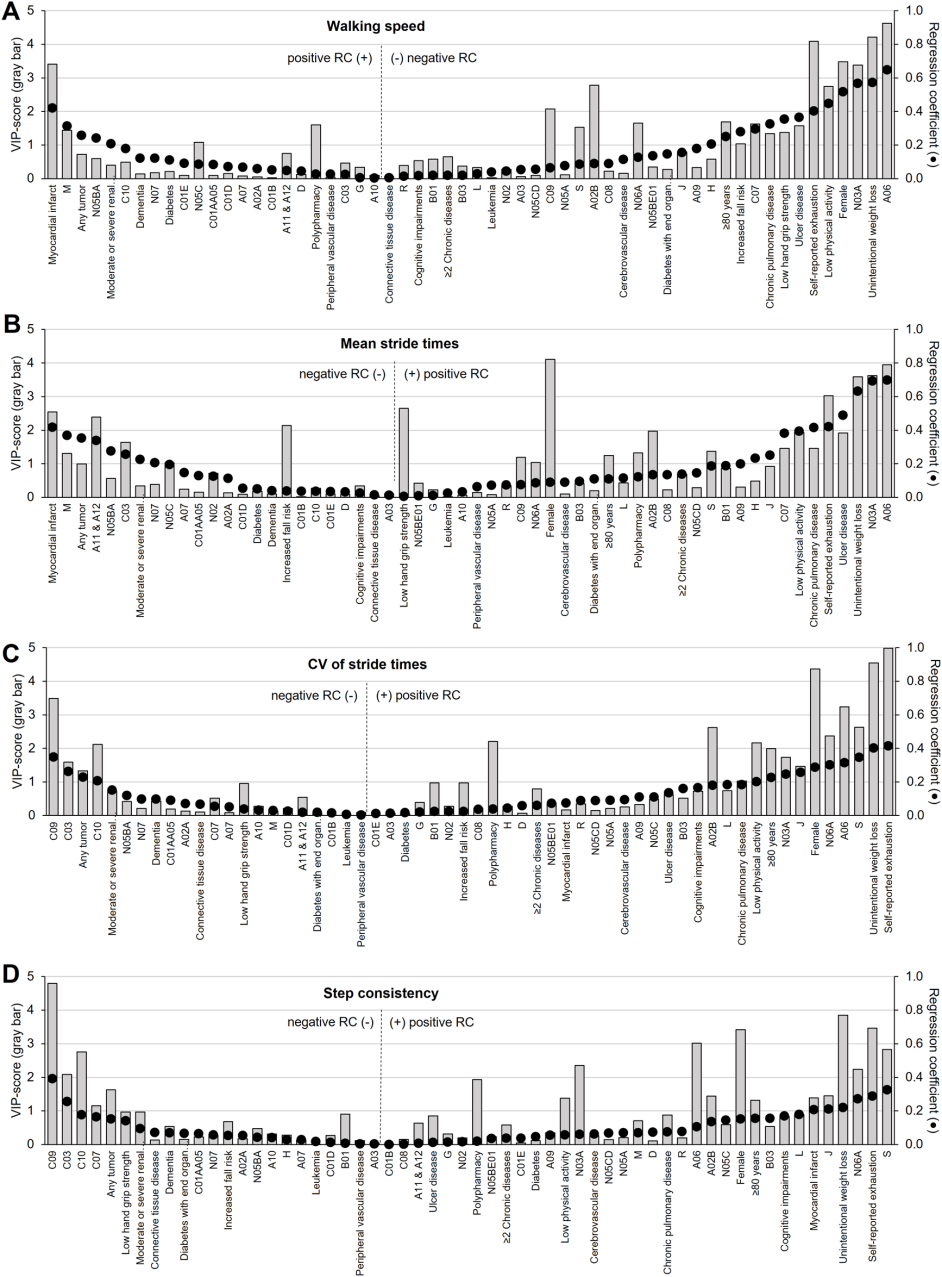
VIP-values and regression coefficients. VIP-values (gray bars; left Y-axis) and regression coefficients (black dots (●); right Y-axis) of all population characteristics, comorbid diseases and medications used are presented for **(A)** walking speed, **(B)** mean stride times, **(C)** CV of stride times, and **(D)** step consistency. The variables are placed on the horizontal axis and sorted according to the height of the regression coefficient. Variables placed at the outer left and right side of the graph have a stronger association with the gait parameter than the variables in the middle of the graph. Variables with a VIP-value of >1 are important to the model. Note that right of the vertical dotted line the variables are presented that are associated with impaired gait ability, that is negative regression coefficients for walking speed, and positive regression coefficients for mean stride times, CV of stride times and step consistency. See Table 1 for a description of the ATC-drug classes.

In Fig 1, the VIP-values and regression coefficients of all population characteristics, comorbid diseases and medication-use for the gait parameters are presented. The most important variables to the model (highest VIP-values) were unintentional weight loss, self-reported exhaustion, being female, and the use of drugs for constipation (A06). These variables were associated with lower walking speed (negative regression coefficients), and higher mean and CV of stride times and less consistent steps (positive regression coefficients).

**Fig 2:**
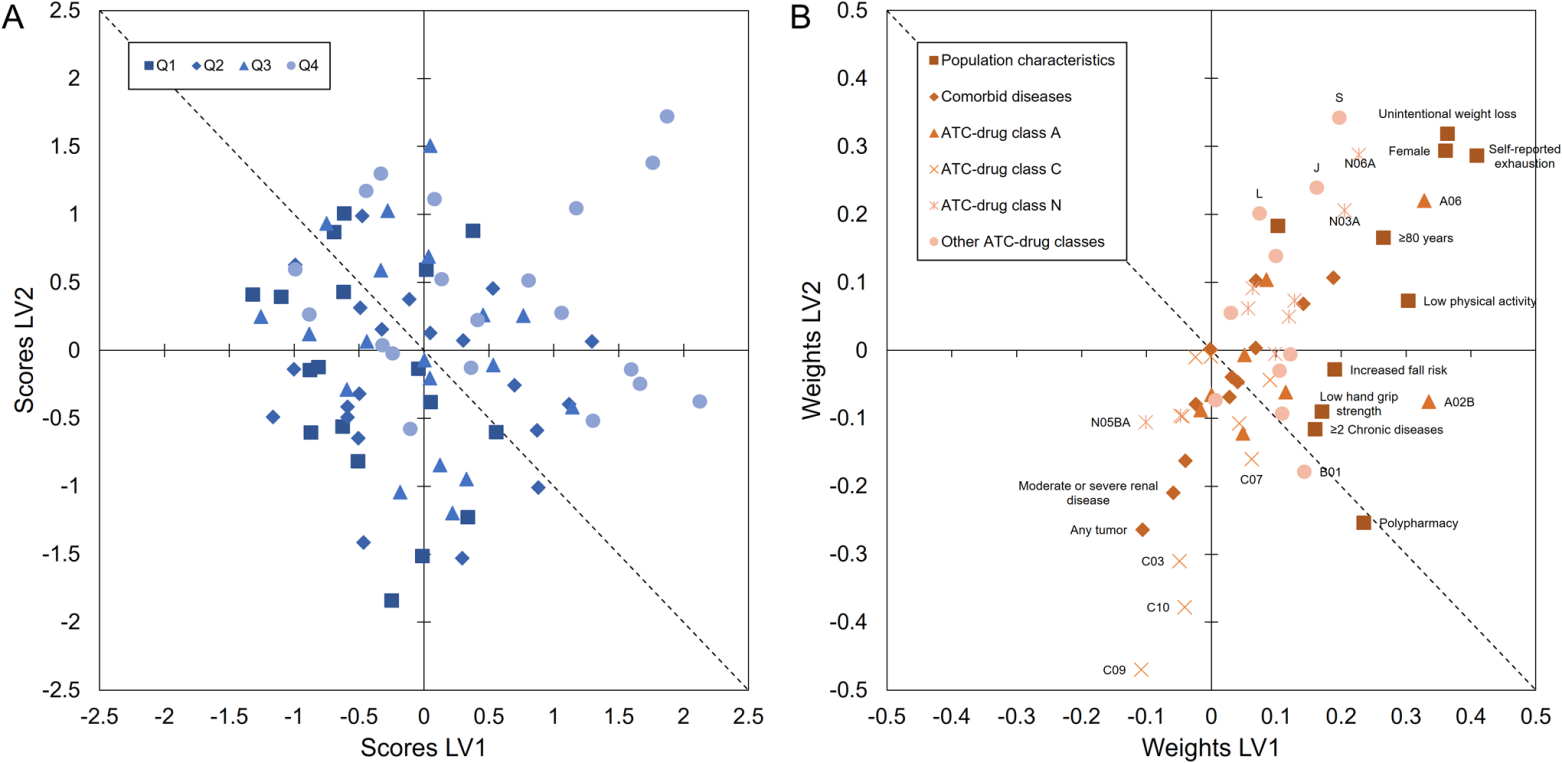
Score and weight plot. The score-plot **(A)** shows the relationship between the participants. The participants are categorized according to their CV of stride times: quartile 1 (Q1; lowest variability; < 2.6%), quartile 2 (Q2; 2.7–3.4%), quartile 3 (Q3; 3.4–4.4%), and quartile 4 (highest variability in stride times; >4.4%). Coupled to the weight plot **(B)**, the inter-relatedness among the patient characteristics and stride variability is revealed. The weight plot can be considered as a coordinate system, where the underlying structure between the variables in relation to stride variability is revealed. The most influential variables are situated far from origo, with the variables at upper right quadrant associated with higher stride variability, and those situated in the lower left quadrant are associated with lower variability.

The association between the participants and the independent variables (population characteristics, comorbid diseases, and medication-use) in relation to the gait parameters is revealed in Fig 2. The position of the participants in a given direction in the score plot (Fig 2A) is influenced by the independent variables lying in the same direction in the weight plot (Fig 2B). Participants in the upper right quadrant of the score plot had overall higher stride variability, whereas persons clustered in the lower left quadrant had lower stride variability. Furthermore, in the weight plot, the relations between the independent variables are visualized. Variables with high regression coefficients and high VIP-values, e.g., unintentional weight loss, self-reported exhaustion, and being female (see Fig 1), are situated in the upper right quadrant of the weight plot, illustrating that they are associated with each other (as can be also seen by the captured variance of LV1 as presented in Table 1), and are associated with higher values for the gait parameters (higher regression coefficients; see Fig 1).

**Fig 3:**
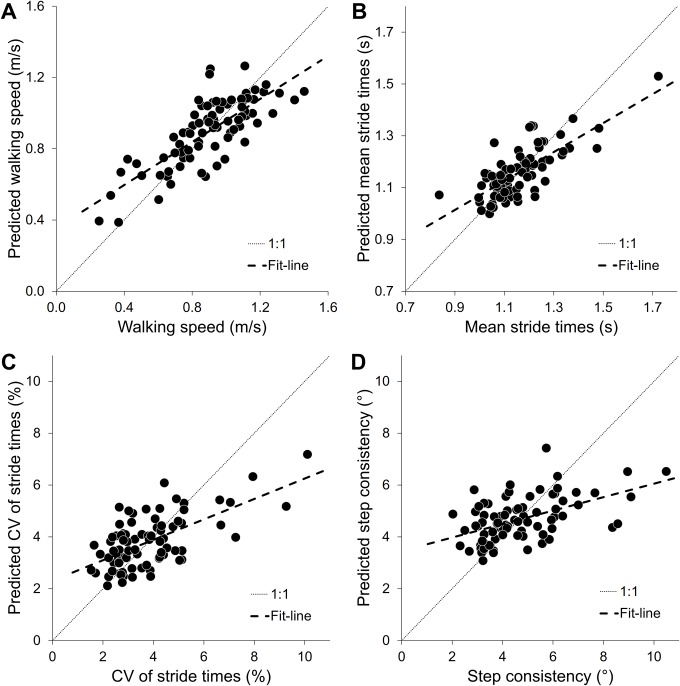
Observed versus predicted values. For **(A)** walking speed, **(B)** mean stride times, **(C)** CV of stride times, and **(D)** step consistency the observed and predicted values are presented. The striped line represents the fit-line, and the dotted line is the 1:1-line.

The fit and predictive ability of the model are illustrated in Fig 3. The observed versus predicted values of the PLS model for walking speed (R^2^ = 60%; Q^2^ = 92%), mean stride times (R^2^ = 56%; Q^2^ = 98%), CV of stride times (R^2^ = 39%; Q^2^ = 88%), and for step consistency (R^2^ = 26%; Q^2^ = 87%) are presented in this figure.

In summary, the results show that impairments in gait performance are strongest associated with unintentional weight loss, self-reported exhaustion, being female and the use of laxatives (A06). These variables had overall most variance captured by the model, and the highest VIP-values and regression coefficients.

## Discussion

In the present cross-sectional study, we aimed to determine the association between population characteristics, comorbid diseases, medication-use and gait parameters in a population of older patients visiting a geriatric outpatient clinic. Therefore, a PLS-regression model was created testing the association between the use of medications in various ATC-drug classes, comorbid diseases, and common factors in a geriatric population, versus gait parameters. In total, 58 variables were included, explaining 45% of the total variance in four gait parameters, namely walking speed, mean stride times, variation in stride times, and step consistency. R^2^- and Q^2^-values showed that the model had an appropriate fit and a good predictive ability (see Fig 3).

We created a statistical model including characteristics common in a population of older patients visiting a geriatric outpatient clinic. 45% of the variance in four gait parameters was explained by this model. Gait is a complex motor behavior requiring adequate integration of sensor-motor information. With aging, there is an age-related deterioration of postural control due to progressive loss of functioning of the neurophysiological system, resulting in, amongst others, visual problems, vestibular impairment, affected proprioception, changes in central processing mechanisms, reduced muscle strength, and slower reaction times [37,38]. These factors are also present in our geriatric study population, but were not as such included in our model. Therefore, these not included aging-related factors presumably account for the unexplained variance in gait parameters in our model.

Nevertheless, most variance in the gait variables was explained by the first LV, and the clustered variables in this LV were therefore strongest related with the gait parameters. In particular, frailty-related criteria were clustered in this first LV, such as unintended weight loss, self-reported exhaustion, low physical activity, and low hand grip strength (see Table 1 and Fig 2), and these were associated with lower walking speed, and higher mean stride times, increased variability of stride times, and less consistent steps. The identified association between these frailty-related indicators and gait parameters is in line with previous research [15,39]. Montero-Odasso et al. [15] suggested that frailty reflects a general deterioration in the neurophysiological system. At the same time, gait can be considered as a complex task that is highly regulated by neurophysiological systems. Consequently, the decreased walking speed, increased stride variability, and worse step consistency that was seen in frail elderly and was found to be related with frailty-related parameters as found in the present study, may reflect the multisystem reduction in neurophysiological capacity.

Based on a previous literature review [8], we anticipated a strong association between the use of psychotropic drugs and gait performance. However, the association of the use of antiepileptics (N03A) and antidepressants (N06A) with impaired gait ability was moderate, while hardly any association with other psychotropic drug classes was observed. We suggest that the effect of the deterioration in physical functioning in the frailest elderly is so large, that the added impact of the side-effects of psychotropics on gait regulation is relatively small. What also may have contributed to the weak associations between gait performance and psychotropic drugs is that the dosages of drugs that were taken by our study population (see S1 Table) was much lower than reported in the aforementioned literature review [8]. This underpins the recommendation of Lader et al. [40] that psychotropic drugs—when administered—should only be prescribed at the lowest effective dose and for a restricted period of time to limit the side-effects of these drugs.

Beside the strong association between frailty-related characteristics and gait parameters, and the moderate association of psychotropic drugs with gait, in the present study also other medication classes were found to be associated with impaired gait ability. The use of laxatives (A06), agents acting on sensory organs (ATC-class S), and drugs for peptic ulcer and reflux disease (A02B) were strongly associated with lower walking speed and higher stride variability as was indicated by high VIP-values and regression coefficients (Fig 1), and illustrated in the score and weight plots (Fig 2). These medication-classes are among the most prescribed medications, and are much more commonly used in individuals with indicators of frailty. For instance, laxatives are used for constipation that, in turn, may be caused by inactivity (one of the indicators of frailty) or the use of constipation-associated medications (e.g., opioid analgesics) [41]. Therefore, these kind of drugs may act as frailty markers [42,43].

The results of the present study correspond with a study of Askari et al. [43] who reported that besides known “fall-risk-increasing drugs”, such as analgesics (N02), anti-Parkinson drugs (N04) and psychoanaleptics (N06), also relatively new classes showed significant association with recurrent falls in elderly, namely nasal preparations (R01), ophthalmologicals (S01), and drugs for acid-related disorders (A02). Especially the finding that drugs for acid-related disorders were associated with recurrent falling and increased stride variability is interesting, because these drugs are commonly used in our study population (see S1 Table) and in the older population in general [44]. Proton-pump inhibitors (PPIs; A02BC), a subclass of drugs for acid-related disorders (A02), have been associated with an increased risk of fractures [45]. It is assumed that PPIs reduce calcium absorption, possibly leading to reduced bone mineral density that would lead to increased fracture risk [46]. Another explanation could be that PPI-use may lead to muscle weakness and gait disorders [46]. This might explain our finding that these drugs were associated with impaired gait ability, and with recurrent falling as Askari et al. [43] found. However, further longitudinal research is necessary to confirm this suggestion.

## Conclusion

In the present study, we found an association between frailty-related parameters and impaired walking ability, as was expressed by lower walking speed and increased stride variability. The absence of a strong association between the use of psychotropic drugs and gait parameters is in accordance to our hypothesis that results from studies in healthy and relatively young elderly cannot be extrapolated to a frail population, because comorbid diseases and the use of many medications are complicating factors in this population leading to multisystem reduction in the neurophysiological system. Therefore, in future studies among frail elderly we recommend to develop models which include frailty-related parameters.

## Supporting Information

S1 Appendix Detailed mathematical description of the PLS regression analysis. (PDF)

S1 Dataset Dataset containing the prevalence of the population characteristics, comorbid diseases, and medication-use per ATC-drug class (as listed in Table 1), and the raw values of the gait parameters. (XLS)

S1 Table Prevalence of the medications used by the study’s participants and the dosage range. (PDF)
