# Supplementary material for: The Association of Medication-Use and Frailty-Related Factors with Gait Performance in Older Patients
Source: PLoS One. 2016 Feb 22;11(2):e0149888. doi: 10.1371/journal.pone.0149888 (PMC4763331; doi:10.1371/journal.pone.0149888)
Supplement: S1 Appendix — (PDF) [file pone.0149888.s001.pdf]

S1 Appendix

Detailed mathematical description of the PLS regression analysis.

PLS – Analysis [1–3]

The *X* and *Y*-matrices represent respectively, the independent variables (population characteristics, co-morbidities and medication-use) and the dependent variables (gait parameters):

$$X = \begin{pmatrix} x_{11} & \cdots & x_{1j} \\ \vdots & \ddots & \vdots \\ x_{i1} & \cdots & x_{ij} \end{pmatrix} \qquad Y = \begin{pmatrix} y_{11} & \cdots & y_{1j} \\ \vdots & \ddots & \vdots \\ y_{i1} & \cdots & y_{ij} \end{pmatrix} \qquad (1)$$

with i is the ith participant and j the jth variable. The relationship between the *X* and *Y* is defined by the function *F*: $Y = F * X + e$, where *F* is modelled with the PLS analysis.

Number of latent variables (LVs) based the goodness of prediction (Q2)

$$Q2_k = 1 - \frac{PRESS_k}{RSS_{k-1}} \qquad (2)$$

$$PRESS = \sum\left(y_{k-1,m} - \hat{y}_{k-1,-m}\right)^2 \qquad (3)$$

where *PRESS* is the predictive sum of squares of the model containing *k* components and *RSS* is the residual sum of squares of the model. The *PRESS* depends on the $y_{k-1,m}$ the residual of observation *m* when *k*–1 components are fitted in the model and $\hat{y}_{k-1,-m}$ the predicted *y* when the latest observation of *m* is removed. When Q2 decreases after reaching a plateau, this is considered the optimal number of latent variables.

Goodness of fit

The R2 explains how well the model fits the data and is defined by te residual sum of squares (RSS) of the kth LV and the total sum of squares (TSS):

$$R2_k = 1 - \frac{RSS_k}{TSS} \qquad (4)$$

The scores

Scores of the PLS reflect the individual participants contribution/position on the LVs as follows:

$$X = T * P' + xres \text{ and } Y = U * Q' + yres \qquad (5)$$

X represents the independent variables (population characteristics, co-morbidities and medication-use), with *T* are the *X*-scores, *P* the *X*-loadings, *U* the Y-scores, and *Q* as *Y*-loadings.

X-weights (W)

Weights describe the importance of the variables on the model for individual latent factors, if they are for all identified LVs near zero than they add little to the model.

Weights are defined by the *X*-loadings (*P*) and the matrix of weights from the model (see eq. 6). They represent the correlation between the *X*-variables and *U*, whereas *Q* represents the correlation between the *Y*-variables and *T* (see eq. 5). Note that the *X*-loadings *P* and the *X*-weights *W* are very similar.

$$W^* = (P * w)^{-1} \qquad (6)$$

The Variable Importance of Projection (VIP)

The VIP-values are based on the explained sum of squares and the weights as follow:

$$VIP_j = \sqrt{p \sum_{k=1}^{N} \left[SS_k \left(w_{kj}/\|w_k\|^2\right)\right] / \sum_{k=1}^{N} (SS)_k} \qquad (4)$$

with SS_k is the explained sum of squares of the kth LV, *N* the number of LVs in the model. The VIP_j weights w_{kj} quantify the contribution of each variable *j* according to the variance explained by each kth LV.

References

1. Boulesteix A-L, Strimmer K. Partial least squares: a versatile tool for the analysis of high-dimensional genomic data. Brief Bioinform. 2007;8: 32–44. doi:10.1093/bib/bbl016
2. Eriksson L, Johansson E, Kettaneh-Wold N, Trygg J, Wikström C, Wold S. Multi- and megavariate data analysis. Part I - Basic principles and applications. Umea, Sweden: Umetrics; 2006.
3. Abdi H. Partial least squares regression and projection on latent structure regression (PLS regression). WIREs Comp Stat. 2010;2: 97–106. doi:10.1002/wics.051
