# Supplementary material for: The Association of Medication-Use and Frailty-Related Factors with Gait Performance in Older Patients
Source: PLoS One. 2016 Feb 22;11(2):e0149888. doi: 10.1371/journal.pone.0149888 (PMC4763331; doi:10.1371/journal.pone.0149888)
Supplement: S1 Table — (PDF) [file pone.0149888.s003.pdf]

S1 Table

Table S1. Prevalence of the medications used by the study's participants and the dosage range.

ATC-code	Description	*n*	(%)	Dosage range	
A02A	*Antacids*	2	(3%)		
A02AA02	magnesium oxide	2	(3%)	724-1448	*mg*
A02B	*Drugs for peptic ulcer and gastro-oesophageal reflux disease (GORD)*	31	(39%)		
A02BA02	ranitidine	3	(4%)	150-300	*mg*
A02BC01	omeprazole	12	(15%)	20-80	*mg*
A02BC02	pantoprazole	11	(14%)	20-40	*mg*
A02BC03	lansoprazole	1	(1%)	15	*mg*
A02BC04	rabeprazole	1	(1%)	20	*mg*
A02BC05	esomeprazole	3	(4%)	40-80	*mg*
A03	*Drugs for functional gastrointestinal disorders*	3	(4%)		
A03AA04	mebeverine	1	(1%)	400	*mg*
A03FA01	metoclopramide	1	(1%)	10	*mg*
A03FA03	domperidone	1	(1%)	30	*mg*
A06	*Drugs for constipation*	12	(15%)		
A06AC01	ispaghula (psylla seeds)	3	(4%)	1-3	*dose/day*
A06AD65	macrogol, combinations	9	(11%)	1-2	*mg*
A06AD11	lactulose	2	(3%)	15	*ml*
A07	*Antidiarrheals, intestinal antiinflammatory/antiinfective agents*	2	(3%)		
A07DA03	loperamide	1	(1%)	2	*mg*
A07EC02	mesalazine	1	(1%)	1000	*mg*
A09	*Digestives, incl enzymes*	1	(1%)		
A09AA02	multienzymes (lipase, protease, etc.)	1	(1%)	3	*dose/day*
A10	*Drugs used in diabetes*	11	(14%)		
A10AD05	insulin aspar	1	(1%)	32	*units*
A10BB01	glibenclamide	1	(1%)	7.5	*mg*
A10BB03	tolbutamide	2	(3%)	1000	*mg*
A10BB12	glimepiride	3	(4%)	0.5-2	*mg*
A10BD02	metformin and sulfonylureas	6	(8%)	1000-2550	*mg*

ATC-code	Description	*n*	(%)	Dosage range	
A11&A12	*Vitamins & mineral supplements*	25	(31%)		
A11BA	multivitamins, plain	4	(5%)		
A11CC03	alfacalcidol	3	(4%)	0.25	*μg*
A11CC05	colecalciferol	16	(20%)	400-880	*IE*
A11DA01	thiamine (vit B1)	2	(3%)	100	*mg*
A12AA02	calcium glubionate	1	(1%)	500	*mg*
A12AA04	calcium carbonate	15	(19%)	500	*mg*
B01	*Antithrombotic agents*	34	(43%)		
B01AA07	acenocoumarol	9	(11%)	1	*g*
B01AC04	clopidogrel	4	(5%)	75	*mg*
B01AC06	acetylsalicylic acid	3	(4%)	80	*mg*
B01AC07	dipyridamole	4	(5%)	200-400	*mg*
B01AC08	carbasalate calcium	22	(28%)	38-100	*mg*
B03	*Antianemic preparations*	4	(5%)		
B03AA02	ferrous fumarate	1	(1%)	600	
B03AA07	ferrous sulfate	1	(1%)	325	*mg*
B03BA03	hydroxocobalamin	1	(1%)	1	*dose/day*
B03BB01	folic acid	3	(4%)	5	*mg*
*C01AA05**	*Digoxin*	4	(5%)		
C01AA05	digoxin	4	(5%)	0.0625-0.25	*mg*
C01B	*Antiarrhythmics (class I and III), excl. type IA*	2	(3%)		
C01BC04	flecainide	2	(3%)	100	*mg*
*C01BA**	*Type IA antiarrhythmics**	0	(0%)		
C01D	*Vasodilators used in cardiac diseases*	10	(13%)		
C01DA02	glyceryl trinitrate	2	(3%)		
C01DA08	isosorbide dinitrate	2	(3%)	2.5-5	*mg*
C01DA14	isosorbide mononitraat	8	(10%)	30-100	*mg*
C01DX16	nicorandil	1	(1%)	10	*mg*

ATC-code	Description	n	(%)	Dosage range
C01E	*Other cardiac preparations*	2	(3%)	
C01EB17	ivabradine	2	(3%)	10-70 *mg*
*C03**	*Diuretics*	30	(38%)	
C03AA03	hydrochlorothiazide	17	(21%)	12.5-25 *mg*
C03BA04	chlortalidone	1	(1%)	12.5 *mg*
C03CA01	furosemide	8	(10%)	20-40 *mg*
C03CA02	bumetanide	2	(3%)	1 *mg*
C03DA01	spironolactone	4	(5%)	25-50 *mg*
C03DB02	triamterene	6	(8%)	50 *mg*
C03EA03	epitizide and potassium-sparing agents	2	(3%)	4 *mg*
C07	*Beta blocking agents*	25	(31%)	
C07AA07	sotalol	1	(1%)	240 *mg*
C07AB02	metoprolol	21	(26%)	25-200 *mg*
C07AB03	atenolol	2	(3%)	25-100 *mg*
C07AB04	acebutolol	1	(1%)	100 *mg*
C07AG02	carvedilol	1	(1%)	12.5 *mg*
C08	*Calcium channel blockers*	13	(16%)	
C08CA01	amlodipine	7	(9%)	5-10 *mg*
C08CA02	felodipine	1	(1%)	5 *mg*
C08CA05	nifedipine	4	(5%)	30-240 *mg*
C08DA01	verapamil	1	(1%)	120 *mg*
C09	*Agents acting on the renin-angiotensin system*	31	(39%)	
C09AA02	enalapril	5	(6%)	5-40 *mg*
C09AA03	lisinopril	5	(6%)	10-20 *mg*
C09AA04	perindopril	7	(9%)	2-8 *mg*
C09AA06	quinapril	1	(1%)	20 *mg*
C09CA01	losartan	4	(5%)	50-100 *mg*
C09CA03	valsartan	1	(1%)	80 *mg*
C09CA04	irbesartan	4	(5%)	150-300 *mg*
C09CA06	candesartan	4	(5%)	16-20 *mg*

ATC-code	Description	*n*	(%)	Dosage range	
C10	*Lipid modifying agents*	38	(48%)		
C10AA01	simvastatin	26	(33%)	10-40	*mg*
C10AA03	pravastatin	4	(5%)	20-40	*mg*
C10AA04	fluvastatin	1	(1%)	80	*mg*
C10AA05	atorvastatin	4	(5%)	20-40	*mg*
C10AA07	rosuvastatin	2	(3%)	5-40	*mg*
C10AB04	gemfibrozil	1	(1%)	1200	*mg*
C10AX09	ezetimibe	1	(1%)	10	*mg*
D	*Antifungals for dermatological use*	1	(1%)		
D02AB	zink products	1	(1%)	1	
G	*Genito urinary system and sex hormones*	11	(14%)		
G01AF04	miconazole	1	(1%)	1	*dose/day*
G01AF15	butoconazole	1	(1%)		
G03CA04	estriol	2	(3%)	0.5	*mg*
G03HA01	cyproterone	1	(1%)	50	*mg*
G04BD08	solifenacin	2	(3%)	5	*mg*
G04BD10	darifenacin	1	(1%)	7.5	*mg*
G04CA01	alfuzosin	1	(1%)	10	*mg*
G04CA02	tamsulosin	5	(6%)	0.4-0.5	*mg*
G04CB01	finasteride	1	(1%)	5	*mg*
G04CB02	dutasteride	2	(3%)	0.4-0.5	*mg*
H	*Systemic hormonal preprarations, excl. Sex hormones and insulins*	5	(6%)		
H03AA01	levothyroxine sodium	5	(6%)	50-150	*µg*
J	*Antiinfectives for systemic use*	4	(5%)		
J01XE01	nitrofurantoin	3	(4%)	50-100	*mg*
J04BA02	dapsone	1	(1%)	100	*mg*
L	*Antineoplastic and immunomodulating agents*	1	(1%)		
L01BC02	fluorouracil	1	(1%)		

ATC-code	Description	*n*	(%)	Dosage range	
M	*Musculo-skeletal system*	10	(13%)		
M04AA01	allopurinol	4	(5%)	100-200	*mg*
M01AB05	diclofenac	1	(1%)	100	*mg*
M05BA04	alendronic acid	4	(5%)	70	*mg/week*
M05BA06	ibandronic acid	1	(1%)	150	*mg/month*
M09AA01	hydroquinine	2	(3%)	200	*mg*
N02	*Analgesics (no paracetamol)*	5	(6%)		
N02AX02	tramadol	2	(3%)	37.5-50	*mg*
N02BA01	acetylsalicylic acid	3	(4%)	80	*mg*
N02BE	*Paracetamol/acetaminophen*	9	(11%)		
N02BE01	buspirone	5	(6%)	500-1500	*mg*
N02BE51	paracetamol, combinations excl. psycholeptics	1	(1%)	65	*mg*
N02BE71	paracetamol, combinations with psycholeptics	3	(4%)	10-30	*mg*
*N03A**	*Antiepileptics*	6	(8%)		
N03AE01	clonazepam	2	(3%)	0.5-1	*mg*
N03AF01	carbamazepine	1	(1%)	400	*mg*
N03AG01	valproic acid	2	(3%)	600-1600	*mg*
N03AX12	gabapentin	1	(1%)	600	*mg*
N03AX16	pregabalin	1	(1%)	150	*mg*
*N05A**	*Antipsychotics*	2	(3%)		
N05AD01	haloperidol	1	(1%)	1	*mg*
N05AX12	aripiprazole	1	(1%)	5	*mg*
*N05BA**	*Anxiolytics (benzodiazepine-derivatives)*	8	(10%)		
N05BA01	diazepam	1	(1%)	6	*mg*
N05BA04	oxazepam	5	(6%)	10-30	*mg*
N05BA12	alprazolam	1	(1%)	1.5	*mg*
N05BAXX	mexazolam	1	(1%)	0.5	*mg*

ATC-code	Description	n	(%)	Dosage range	
N05C	*Other hypnotics, excl. benzodiazepine-derivatives*	7	(9%)		
N05CH01	melatonin	3	(4%)	1-2	*mg*
N05CM09	valerianae radix	3	(4%)	45-350	*mg*
N05CX04	clomethiazole, combinations	1	(1%)	20	*mg*
*N05CD**	*Hypnotics & sedatives (benzodiazepine-derivatives)*	5	(6%)		
N05CD02	nitrazepam	1	(1%)	2.5	*mg*
N05CD07	temazepam	3	(4%)	5-10	*mg*
N05CF01	zopiclone	1	(1%)	7.5	*mg*
*N06A**	*Antidepressants*	12	(15%)		
N06AB04	citalopram	3	(4%)	20	*mg*
N06AB05	paroxetine	2	(3%)	20	*mg*
N06AB06	sertraline	2	(3%)	50-100	*mg*
N06AX11	mirtazapine	3	(4%)	30-45	*mg*
N06AX16	venlafaxine	2	(3%	75-450	*mg*
N07	*Other nervous system drugs*	2	(3%)		
N07CA01	betahistine	2	(3%)	8-16	*mg*
R	*Respiratory system*	13	(16%)		
R01AD08	fluticasone	2	(3%)	100	*μg*
R03AC13	formoterol	3	(4%)	200-400	*mg*
R03AK06	salmeterol and fluticason	4	(5%)	1-2	*dose/day*
R03AL01	fenoterol and ipratropium bromide	1	(1%)	4	*dose/day*
R03BA02	budesonide	4	(5%)	2-12	*mg*
R03BB04	tiotropium bromide	7	(9%)	1	*dose/day*
R05CB01	acetylcysteine	1	(1%)	1200	*mg*
R06AE09	levocetirizine	1	(1%)	5	*mg*
R06AX15	mebhydrolin	1	(1%)	1	*dose/day*
R06AX27	desloratadine	2	(3%)	5	*mg*

ATC-code	Description	*n*	(%)	Dosage range	
S	*Sensory organs*	8	(10%)		
S01ED02	betaxolol	1	(1%)	1	*dose/day*
S01ED51	timolol, combinations	1	(1%)	1	*dose/day*
S01EE04	travoprost	2	(3%)	1	*dose/day*
S01XA20	artificial tears and other indifferent preparations	4	(5%)	1	*dose/day*
S02CA03	hydrocortisone and antiinfectives	1	(1%)	1	*dose/day*
